# From Growth to Greatness: A Leading Article on the Professionalisation, Health and Performance Challenges in Women’s Football

**DOI:** 10.1007/s40279-025-02266-7

**Published:** 2025-08-25

**Authors:** Dawn Scott, Ric Lovell, Belinda Wilson

**Affiliations:** 1 FIFA Women’s Football Division, FIFA, Zurich, Switzerland; 2 Faculty of Science, Medicine and Health, University of Wollongong, Wollongong, NSW Australia

## Abstract

The FIFA Women’s World Cup AUS & NZL 2023™ marked a pivotal moment in women’s football, reflecting unprecedented growth in global attention, revenue and participation. This leading article discusses the transformation in women’s football, emphasising the increasing demands on players alongside the sport’s professionalisation. To support this transformation, the FIFA Female Health project is introduced, which seeks to address critical health and performance challenges specific to female players by raising awareness, supporting research and developing education initiatives for women’s football stakeholders. In this prologue, we examine the rise in physical and tactical requirements, driven by more rigorous match schedules, advanced pressing strategies and extended travel demands. Alongside these challenges, we emphasise the growing need for multidisciplinary support teams to address the unique biopsychosocial needs of female footballers. This article calls for further research, education and investment in player support. By examining the interplay between professionalisation and performance, this special issue aims to provide insights into the critical areas where scientific innovation and collaboration are necessary to ensure player wellbeing and sustained performance in the evolving landscape of women’s football.

## Introduction

The FIFA Women’s World Cup 2023™ (WWC), jointly hosted by Australia and New Zealand, marked a watershed moment for women’s sport, setting new records on and off the pitch and signalling a seismic shift in global attention towards women’s football. In her closing remarks, FIFA Chief Women’s Football Officer Sarai Bareman reflected, *"2023 is about showing the world what it means to take the game beyond greatness. To put our players on the pedestal that they belong, to fill the stadiums, smash records, break down barriers and show every young girl and boy, from every corner of the world that they can dream to make a living from football."* [1]. This statement encapsulates the evolving narrative around women’s football and its growing global significance.

However, alongside this rapid professionalisation, new challenges have emerged, particularly concerning the health and performance of female athletes. Recognising these challenges, the FIFA Female Health Project [2] was launched to raise awareness and bridge the knowledge gap for all stakeholders within the women’s game. This initiative, a collaboration between the FIFA Women’s Football Division and Medical Department, emphasises the need to understand the unique biopsychosocial needs of female players and to develop evidence-informed strategies that enhance their health, performance and longevity in the sport.

Aligned with these objectives, this special edition represents a synthesis of the current knowledge and identifies future research directions in key areas of player welfare that remain under-researched or require further application in practice. While investment and visibility in women’s football have increased, gaps persist in workload management amidst rising match intensity and density, injury prevention strategies tailored to female athletes and the physiological effects of the menstrual cycle and hormonal contraceptives on performance. Additionally, disparities in league structures, support systems and access to multidisciplinary expertise continue to shape the player experience, highlighting the need for evidence-informed policies and educational programs that ensure sustainable career development and well-being.

This special issue presents a series of articles that delve into these topics, addressing menstrual cycle tracking, hormonal contraception, nutrition, sleep, recovery, pregnancy, postpartum care and injury risk in women’s football. By advancing knowledge in these areas, it aims to bridge critical research gaps and promote science-driven strategies that not only enhance player welfare and optimise performance, but also contribute to the continued professionalisation of the sport.

## Growth and Professionalisation

This special issue comes at a critical time, where the growth of women’s football is undeniable. The 2023 tournament was unprecedented, with 32 teams and $570 million in revenue, making it the first WWC to break even financially [3]. By the end of the tournament, over 3 billion views of content were registered on FIFA’s social and digital platforms [3]. These milestones are not just records; they underscore a transformation in the sport’s global appeal and its market value. Domestically, leagues such as the Women’s Super League (WSL) in England and the National Women’s Soccer League (NWSL) in the US saw attendance and viewership rise by over 40% in the 2023/24 season, with clubs setting new precedents for player investment and franchise valuations. Indeed, Deloitte projects that global revenues in elite women’s sports will reach £2.35 billion in 2025, a 240% increase in just 4 years [4].

Despite these advances, the path toward full professionalisation remains uneven. Many nations still lack the infrastructure, governance and investment required to support long-term player development and career sustainability. The latest FIFA Benchmarking Report [5] surveyed 211 Member Associations, drawing input from 86 leagues and 669 clubs. A new tiered system (Tiers 1–3) was introduced to classify leagues based on indicators such as league structure, licensing and broadcasting. Marked differences were evident between tiers. In Tier 1, 72% of players reported earning a reliable income from football alone, compared with just 58% and 21% in Tiers 2 and 3, respectively. Players in Tier 1 were also more likely to have written contracts, access superior training and medical facilities and benefit from maternity and childcare support. Conversely, players in lower tiers were more likely to hold dual careers, face limited access to player associations and train in less professional environments. Addressing these disparities will require targeted reforms (e.g. minimum salary standards, maternity protections) alongside sustained investment and policy leadership to foster a more equitable and sustainable global football ecosystem.

## Evolving Physical and Tactical Demands

Accompanying the professionalisation of women's football is a significant rise in both the physical and tactical demands placed on players. The WWC AUS & NZL 2023™ exemplified this evolution, with extended match durations and more teams implementing defined pressing strategies. A key trend was the increased distance covered in the mid-range speed zone (13–19 km•h^−1^), driven by faster recovery runs and heightened tactical responsibilities [6]. This speed zone may represent the efforts required for maintaining defensive and offensive structure, enabling efficient transitions in an increasingly fast-paced and tactically complex game. Moreover, those players at the pinnacle of the professional game have seen a marked increase in the density of matches across both club and national team environments [7]. Elite players accumulate substantial playing time, averaging 304 ± 167 min per 28-day period, alongside significant travel distances of 3742 ± 6430 km within the same timeframe [7]. While evidence on the evolution of player physical capacities remains limited, the increasing frequency and intensity of matches highlight the need for specialised and tailored approaches to player preparation, workload management and recovery strategies to support the well-being and performance of female football players.

## Health and Performance Considerations

In recent years, the burden of injuries has received much media attention, with a number of high-profile players sustaining significant time-loss injuries; recent data suggests a loss of 38.0 days per 1000 h of football [8]. In addition to this, there are other health considerations that are pertinent to female athletes. Some studies have reported that a high proportion of elite and amateur players experience menstrual symptoms that they perceive to inhibit football performance (73–83%) [9, 10]. However, athlete-reported experiences vary widely [11, 12] and objective data remain inconclusive, warranting more rigorous, globally representative research to better understand the effects of menstruation on athletic performance [13, 14]. Notwithstanding, in the absence of pregnancy, post-partum, menstrual dysfunction and hormonal contraception, the cyclical change in concentrations of female sex hormones (primarily oestrogen and progesterone) impact a myriad of physiological and psychological factors, which may ultimately affect readiness to perform [15] [16]. Pelvic health considerations are also significant amongst female athletes, with a 5.45 times greater incidence of urinary incontinence in female athletes than males [17]. FIFPRO have recently outlined minimum standards for player support during pregnancy and motherhood [18]. However, further guidelines providing specific training and support are still required across all aspects of female health, emphasising the need for more translational research. In order for this to be globally appropriate, a culturally sensitive approach must be considered acknowledging potential challenges related to resources, existing beliefs and/or communication [12].

## Multidisciplinary Support

In line with the global professionalism of women’s football, the composition of multidisciplinary support teams may warrant further consideration. Tracking and monitoring physical, psychological and health characteristics is a key part of optimising performance while also protecting the health and well-being of players. However, standardised practices for data collection, data analysis and interpretation are underdeveloped, highlighting a key research priority [19] [20]. Given the multifaceted factors influencing player readiness (e.g. nutrition, sleep, training load, travel, psychological stress, female health status, training status, training age), a multi-disciplinary approach to data interpretation is essential. Whilst support teams in women’s football may benefit from expertise in areas such as endocrinology, pelvic health and menstrual cycle considerations [21], multidisciplinary team composition may be tailored based on competitive level, cultural considerations, available resources and specific player needs. The most recent benchmarking report published by FIFA found less than half of clubs have a sports scientist, nutritionist, mental performance/psychologist, and only 9–16% have access to a female health specialist, clearly highlighting the need for further investment in the women’s game [5]. Indeed, several injury surveillance studies have suggested that the high incidence of injury severity and burden observed could be due to the lack of appropriate staffing available to players [22] [23] [24], albeit direct causal relationships remain unconfirmed. A concerted effort needs to be placed on 1) identifying key support team roles that best support female athletes’ performance and health; 2) advancing research and translating this to accessible training modules with appropriate qualifications; and 3) providing guidance for data collection, analysis and interpretation in female football.

## Academic Interest in Women’s Football

With the growing participation and visibility of women’s football, academic interest in the field has expanded considerably [21]. While research historically lagged behind that of the men’s game [25], recent years have seen encouraging momentum, with increased attention to sex-specific considerations in training methodologies, injury prevention and performance optimisation. As this evidence base continues to grow, it is also important to recognise that many foundational principles of athlete health and performance, such as adaptation to training load, adequate fuelling and safe return-to-play protocols, are sex-agnostic. Acknowledging both the unique biopsychosocial needs of female footballers and the shared foundations of human physiology ensures that research in women’s football can contribute meaningfully. Despite these recent advances, significant disparities remain, particularly in underrepresented topics relevant to female players [26]. Continued investment in rigorous, practically relevant research is essential not only to bridge these data gaps, but also to facilitate the translation of findings into applied settings, ensuring that female-specific evidence informs training, competition and player welfare strategies at all levels of the game [21] [27] [28].

The effective translation of academic research into football practice relies not only on its dissemination, but also on the capacity of multi-disciplinary support teams to implement evidence-informed strategies tailored to the needs of female footballers [21]. Given the significant sex differences, practitioners should critically evaluate traditional research and practices, many of which have historically defaulted to male physiology, while also recognising shared principles of human adaptation that underpin effective preparation for all athletes. Instead, an inclusive and nuanced understanding that incorporates foundational principles of health and performance and female-specific physiological, endocrinological and health considerations (e.g. menstruation, pelvic and breast health) is essential for the optimal physical preparation of female footballers.

Barriers to communication about female health persist in football, often due to limited education among players, coaches and support staff [29, 30]. Furthermore, the global gender gap in coaching remains evident, with only 12 of the 32 head coaches at the WWC AUS & NZL 2023™ being women. The latest FIFA Benchmarking report [5] highlights this disparity further, revealing that women occupy only 22% of head coaching roles in women’s football globally. This under-representation may contribute to communication challenges in female-specific health and performance domains. Nevertheless, coaches are eager to expand their knowledge [31], presenting an opportunity to better integrate emerging research into sports medicine and coaching curricula. Educating multidisciplinary teams through continuing professional development and open-access certification programmes will empower them to deliver evidence-informed support that optimises both the health and performance of female footballers.

## A Call to Action: Investing in the Future of Women’s Football

This special issue not only contributes to the growing body of knowledge on women’s football but also serves as a call to action for researchers, practitioners and governing bodies to continue investing in the future of the game. There is an urgent need for targeted research, education and multi-disciplinary support to address the unique health and performance challenges faced by female players. The FIFA Female Health Project [2] provides a critical foundation, yet further investment and collaboration is needed to ensure that female-specific evidence informs practice at all levels of the game. We emphasise the importance of collaboration between researchers, practitioners and governing bodies to translate findings into actionable strategies. The following articles provide an overview of current understanding about how to support players in areas such as menstrual cycle tracking, sleep, nutrition, recovery, pregnancy and postpartum care. This special issue aims to spark further innovation and collaboration, laying the groundwork for the future of the sport tailored to the needs of female athletes. Together, we can ensure that women’s football continues its trajectory beyond greatness, driven by evidence-informed practices that prioritise the health and performance of its players. 

## Declarations

### Equity, Diversity and Inclusion statement

Our author team consists of two female and one male author; representing early to mid-career researchers working in elite and grassroots football applied practice, governance and education, living in three different countries over two continents.
